# *Streptomyces* BAC Cloning of a Large-Sized Biosynthetic Gene Cluster of NPP B1, a Potential SARS-CoV-2 RdRp Inhibitor

**DOI:** 10.4014/jmb.2205.05036

**Published:** 2022-06-13

**Authors:** Ji-Hee Park, Heung-Soon Park, Hee-Ju Nah, Seung-Hoon Kang, Si-Sun Choi, Eung-Soo Kim

**Affiliations:** 1 Department of Biological Sciences and Bioengineering, Inha University, Incheon 22212, Republic of Korea; 2 Department of Biological Engineering, Inha University, Incheon 22212, Republic of Korea

**Keywords:** Nystatin-like polyene, biosynthetic gene cluster, bacterial artificial chromosome, SARS-CoV-2 RdRp

## Abstract

As valuable antibiotics, microbial natural products have been in use for decades in various fields. Among them are polyene compounds including nystatin, amphotericin, and nystatin-like *Pseudonocardia* polyenes (NPPs). Polyene macrolides are known to possess various biological effects, such as antifungal and antiviral activities. NPP A1, which is produced by *Pseudonocardia autotrophica*, contains a unique disaccharide moiety in the tetraene macrolide backbone. NPP B1, with a heptane structure and improved antifungal activity, was then developed via genetic manipulation of the NPP A1 biosynthetic gene cluster (BGC). Here, we generated a *Streptomyces* artificial chromosomal DNA library to isolate a large-sized NPP B1 BGC. The NPP B1 BGC was successfully isolated from *P. autotrophica* chromosome through the construction and screening of a bacterial artificial chromosome (BAC) library, even though the isolated 140-kb BAC clone (named pNPPB1s) lacked approximately 8 kb of the right-end portion of the NPP B1 BGC. The additional introduction of the pNPPB1s as well as co-expression of the 32-kb portion including the missing 8 kb led to a 7.3-fold increase in the production level of NPP B1 in *P. autotrophica*. The qRT-PCR confirmed that the transcription level of NPP B1 BGC was significantly increased in the *P. autotrophica* strain containing two copies of the NPP B1 BGCs. Interestingly, the NPP B1 exhibited a previously unidentified SARS-CoV-2 RNA-dependent RNA polymerase (RdRp) inhibition activity in vitro. These results suggest that the *Streptomyces* BAC cloning of a large-sized, natural product BGC is a valuable approach for titer improvement and biological activity screening of natural products in actinomycetes.

## Introduction

Actinomycetes are a source for discovery of important secondary metabolites containing a number of drugs and analogs that have been commercialized and are still used in human/animal health and crop protection [1, 2]. Actinomycetes-produced natural products (NPs) are associated with diverse biosynthetic gene clusters (BGCs). BGCs are groups of genes responsible for producing specialized metabolites [3]. The majority of these actinomycetes NP BGCs belong to three types: polyketide synthase (PKS), non-ribosomal peptide synthase (NRPS), and a combination of PKS and NRPS [4]. Among them, some polyketides made by PKS complete the final structure after further modification by enzymes, such as P450 hydroxylase and glycosyltransferase [5, 6]. The representative polyene macrolide antibiotics synthesized by type I PKS include amphotericin, nystatin, candicidin, and NPP, which are generally antifungal compounds with 3–8 conjugated bonds to the core macrolactone rings with 20–40 carbon atoms [7, 8].

A rare actinomycete, *Pseudonocardia autotrophica*, produces NPP A1. This compound has the same core macrolactone structure as nystatin A1, but unlike nystatin A1, it contains a unique disaccharide. NPP A1 has high solubility and low hemolytic activity compared to nystatin A1, but its antifungal activity is lower than that of nystatin A1 [9]. Therefore, NPP B1, which has a heptaene structure from the core macrolactone structure of tetraene, was developed by substituting two site-specific amino acids in module 5 of the NPP A1 biosynthetic gene *nppC* [10]. NPP B1 was developed by genetic manipulation and is less toxic but more powerful as an antifungal drug with similar efficacy in vivo compared to amphotericin B. Therefore, NPP B1 is a promising candidate for pharmacokinetically improved and less toxic polyene antifungal antibiotics [11, 12]. On the other hand, the production level of NPP B1 was very low compared to the strain producing NPP A1 [10]. Various NPP B1 titer-improvement attempts including overexpression of the pathway-specific regulatory gene (*nppRIV*), deletion of global antibiotic downregulator WblA-ortholog, in situ screening of NPP B1 random mutagenesis, co-culture with *Corynebacterium glutamicum*, and cultivation with xenobiotics such as triclosan were unsuccessful [10]. The noticeable improvement was achieved through deletion of a native 125-kb circular plasmid encoding a competing-type I PKS BGC in *P. autotrophica* wild type, resulting in a 147% increase in the NPP B1 production [10]. The highest production level of NPP B1 ever achieved by *P. autotrophica* strains so far was approximately 31.6 mg/l via synergistic combination of the traditional random mutagenesis and pNPPREG chromosomal integration [11].

Cloning and expression of NP BGCs has become a useful strategy to produce, reactivate, improve, and modify the pathways of NPs present at minute quantities in the original actinomycetes isolates [13-15]. However, efficient cloning and overexpression of an entire NP BGC, often as large as 100 kb or more, remain challenging due to the ineffectiveness of current genetic systems in manipulating large NP BGCs [15-18]. In this study, to increase the production level of NPP B1, its BGC was isolated from *P. autotrophica* and overexpressed in the original NPP B1-producing strain. The isolated NPP B1 BGC from the *P. autotrophica* strain does not have the entire NPP B1 biosynthetic gene cluster, so deficient genes were supplemented by homologous recombination using a vector with the 32-kb gene cluster containing deficient genes. As a result, the production level of NPP B1 was increased compared to the original NPP B1 producer. Moreover, based on the fact that the antiviral activity of amphotericin B against some envelope viruses, such as the human immunodeficiency virus, Japanese encephalitis virus, and rubella virus, a fluorescence-based assay using RdRp (RNA-dependent RNA polymerase) of SARS-CoV-2 was performed to identify the previously unknown antiviral activity of NPP B1 in vitro [19-21].

## Materials and Methods

### Strains and Growth Conditions

*P. autotrophica* KCTC9441 was purchased from the Korean Collection for Type Cultures and used for NPP production. The strain was grown routinely in ISP2 agar [malt extract 10 g (DB Difco, USA), yeast extract 4 g (DB Difco) glucose 4 g (TCI, Japan) and agar 20 g/l (Duksan; Korea)] at 30°C for the sporulation and seed culture. The YEME medium [yeast extract 3 g, peptone 5 g (DB Difco), malt extract 3 g, glucose 10 g, sucrose 340 g/l (Daejung, Korea), and 5 mM MgCl_2_·6H_2_O (Daejung)] was used to produce the NPP derivatives [9]. All Escherichia coli strains were incubated at 37°C in LB medium supplemented with the appropriate antibiotics where needed. The *E. coli* DH5α strain (TRANS, China) and EPI300 were used for DNA cloning and plasmid propagation. *E. coli* ET12567 bearing the RK2-derived helper plasmid pUB307 facilitated the intergeneric triparental conjugation. The conjugation experiments were performed as described previously [22].

### Isolation of NPP B1 Biosynthetic Gene Cluster into pESAC-13

A BAC library using pESAC13-Apramycin (Bio S&T, Canada) was constructed. The vector DNA was digested with BamHI. The partially digested high-molecular-weight DNA was size-selected on 1% (w/v) pulsed-field agarose gels in 0.5X TBE on a CHEF DRIII (Bio-Rad, Canada). Partially digested size-selected DNA fragments were ligated to the vector DNA in a volume of 50 μl with 1X ligase buffer and three units of ligase (USB, Canada) at 14°C for overnight incubation. Two microliters of ligation mix were used to transform 20 μl of *E. coli* DH10B cells (Invitrogen, USA) by electroporation using a CellPorator equipped with a voltage booster (Invitrogen). The cells were then selected on LB medium supplemented with 5% sucrose plus Apramycin by incubation at 37°C overnight. The clones were selected and arrayed in 6 × 384-well plates in a medium containing freezing medium supplemented with 10% (v/v) glycerol and Apramycin. The average insert size was evaluated using randomly selected clones. The miniprep of PAC clones and digestion of PAC DNA with DraI was done according to standard procedure. Pulsed-field gel electrophoresis (PFGE) was conducted to check the insert sizes.

### Production and HPLC Quantification of NPP B1

The *P. autotrophica* strains producing NPP B1 were inoculated in 40 ml ISP2 medium containing the appropriate antibiotics at 30°C and 220 rpm for 72 h. The pre-cultures were added to 400 ml YEME medium in a 2-L flask for 72 h. The culture broth was amended with 20 g of Amberlite XAD16 resin (Sigma Aldrich, USA) after 48 h of cultivation. NPP B1 was extracted from the supernatant with one volume of butanol. The final extracts were loaded onto a column packed with a C18 reversed-phase silica gel (Daiso, Japan) for HPLC analysis [10, 11]. The column was equilibrated with 50% solvent A (0.05 M ammonium acetate) (Samchun, Korea) at pH 6.5 and 50% solvent B (methanol) (Burdick & Jackson, USA). The flow rate was set to 1.0 ml/min using the following conditions: 0–3 min, 50–75% B; 3–30 min, 75–100% B; 30–33 min, 100–50% B; and 33–40 min, 50% B [23].

### Isolation of the Total RNA and Gene Expression Analysis by qRT-PCR

For RNA preparation, *P. autotrophica* GG5036SP, *P. autotrophica* NPP001, and *P. autotrophica* NPP002 were grown for 3 days in YEME medium. The samples were taken at 24 and 48 h. The mycelia were harvested by centrifugation and stored in a deep freezer at -80°C after washing twice with distilled water. An RNeasy Mini Kit (Qiagen, Germany) and a Prime Script 1^st^ Strand cDNA Synthesis Kit (TaKaRa, Japan) were used for RNA isolation and cDNA synthesis. Real-time RT-PCR was performed using TaKaRa TB Green Premix Ex Taq GC (Perfect Real Time) with a Thermal Cycler Dice Real-Time System Single (code TP850) (TaKaRa). The PCR conditions included 35 cycles of 30 s at 95°C, 30 s at 58°C, and 30 s at 72°C. The data were collected at each 72°C step, and melting curve analysis was performed at the default settings of 60–95°C. The primer pairs are presented in Table S1. *rpsO* gene was used as the internal control. The transcripts from four biosynthetic genes, *nppA*, *nppC*, *nppI*, and *nppJ*, and six regulatory genes, *nppRI*-*nppRVI*, were analyzed after 35 PCR cycles.

### Construction of pNPPREG and Introduction into pNPPB1s-Containing Strains

The additional NPP B1 regulatory gene in *P. autotrophica* NPP001 was introduced by replacing the apramycin-resistant gene in the pNPPREG with the hygromycin-resistant gene (named pNPPREG2) using a Quick and Easy BAC Modification Kit (GeneBridges, Germany). The pNPPREG was introduced into Red/ET plasmid-containing *E. coli* EPI300. BAC modification was then performed according to the manufacturer’s guide using PCR to amplify the hygromycin-resistant gene in the *apr^R^*-homologous region. The transformants were selected on a hygromycin-containing LB medium and confirmed by PCR. The additional NPP B1-regulated genes into the heterologous host with pNPPB1s were introduced by substituting the apramycin-resistant gene with the kanamycin-resistant gene (pNPPREG3) using the same method, as described above.

### Construction of 8-kb Right-End Portion of NPP B1 BGC-Containing pSE34 Vector

The entire NPP B1 BGC in heterologous hosts was expressed by cloning the 8-kb right-end portion of NPP B1 BGC into an *oriT*-containing pSE34 vector. The pSE34 with *oriT* was digested with XbaI (TaKaRa) and EcoRI (TaKaRa), and the digested vector was amplified by PCR using the primer pairs, which contained an 8-kb gene homologous region. The 8-kb right-end portion of NPP B1 BGC was amplified by PCR using pNPPREG as a template and cloned into pUC19. The 8-kb fragment was digested with NheI (TaKaRa) and XbaI from pUN8 and cloned into a prepared pSE34 vector using an In-Fusion Cloning Kit (TaKaRa). The vector was then digested with HindIII (TaKaRa), and a kanamycin-resistance gene was introduced using the In-Fusion method. The resulting construct was called pSEN8, which was introduced directly into the heterologous host.

### Fluorescence-Based Activity Assay for SARS-CoV-2 RdRp

The fluorescence emitted was recorded by employing GloMax Discover (Promega, USA) using the excitation and emission filters at 485 and 520 nm. This assay records the synthesis of dsRNA in a reaction using ATP (Sigma Aldrich) as a nucleotide substrate and a poly-U (Sigma Aldrich) molecule as a template using fluorescent dye SYTO 9 (Invitrogen), which binds only to dsRNA [24]. The standard reaction contained 50 mM Tris-HCl (pH 8.0), 5 mM MnCl_2_·4H_2_O (Sigma Aldrich), 50 mM NaCl (Samchun), 4 mM DTT (TaKaRa), 3 mM ATP, 13 μg/ml poly-U, and 0.25 μM SYTO 9. The assay was initiated by adding 1 μg/μl RdRp/NSP7/NSP8 (SARS-CoV-2) Complex (BPS Bioscience, USA), and the fluorescence was recorded over 20 min at 30°C. The reaction was conducted in black, 96-well, flat-bottomed plates. For the compound used in this assay, NPP B1 with a purity of over 80% on HPLC was used, and ampicillin, kanamycin, and amphotericin B were purchased from Sigma Aldrich.

## Results

### BAC Library Construction and Heterologous Expression

**Fig. 1:**
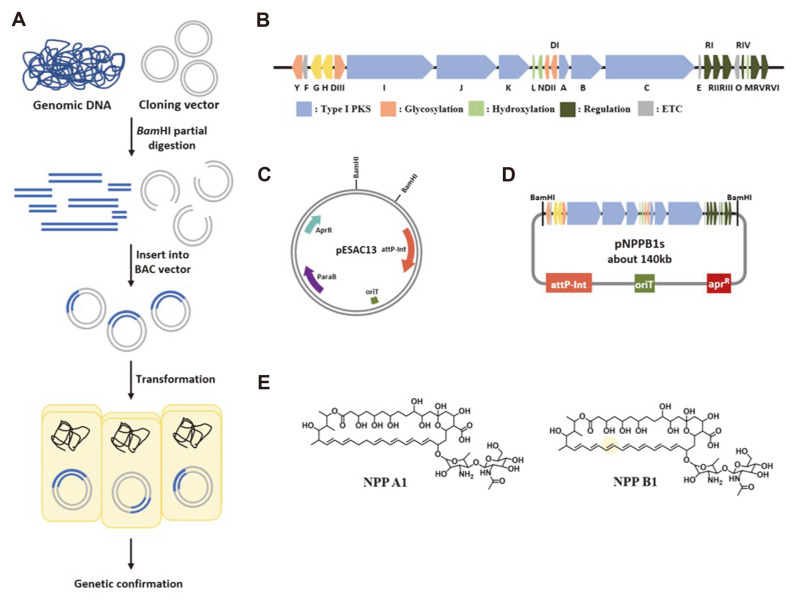
BAC library construction. (**A**) Scheme of the BAC library construction (**B**) NPP biosynthetic gene cluster (**C**) Map of pESAC13 (**D**) Map of pNPPB1s (**E**) Structures of NPP A1 and NPP B1.

The BAC library was constructed by partial digestion with BamHI of genomic DNA to isolate the NPP B1 BGC from *P. autotrophica* GG5036SP. After construction, the BAC library was screened by PCR using *nppY*, *nppN-DII*, and *nppRVI* check primers in NPP B1 BGC. Unfortunately, the entire NPP B1 BGC-containing BAC clone was not screened, but approximately 140 kb of the NPP B1 BGC containing the BAC clone (named pNPPB1s) was isolated. The missing 8-kb right-end portion of the NPP B1 BGC, including *nppO* (encodes for acyl-CoA carboxylase), *nppM* (encodes for ferredoxin), and three regulatory genes (*nppRIV*, *nppRV*, and *nppRVI*) was confirmed by PFGE and sequencing (Fig. 1). An attempt was made to express the pNNPB1s in the heterogeneous hosts, such as *S. lividans* TK21 and *S. coelicolor* M511, using the triparental conjugation method. In contrast with our expectations, no consistent results were observed in this attempt to produce NPP B1 in these heterologous expression systems (data not shown). Therefore, pNPPREG3 was introduced as a homologous recombination to introduce the deficient gene of pNPPB1s to the heterologous hosts with pNPPB1s, but NPP B1 was still not detected. As another method, only 8 kb of the pNPPB1s deficient gene was also introduced in the form of a plasmid using the pSEN8 vector into the heterologous hosts with pNPPB1s, but NPP B1 could not be observed, implying that some unknown gene(s) present in *P. autotrophica* might be required for NPP B1 production in *Streptomyces* heterologous hosts.

### Homologous Overexpression of NPP B1 Biosynthetic Gene Cluster in NPP B1-Producing Strains

**Fig. 2:**
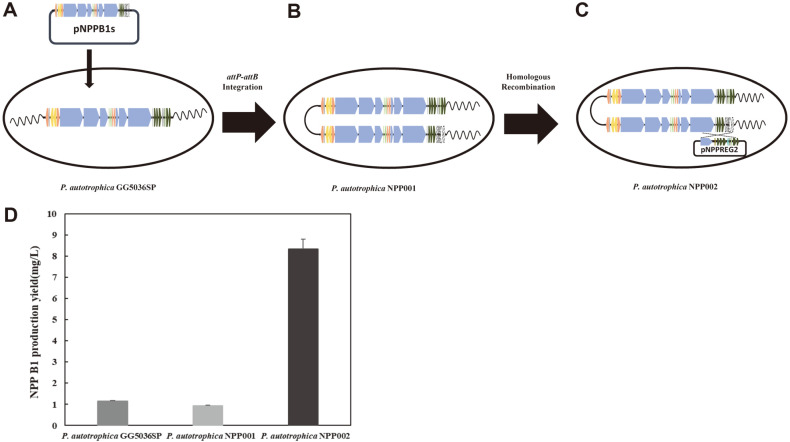
Scheme of homologous overexpression and NPP production yield. (**A**) *P. autotrophica* GG5036SP (**B**) *P. autotrophica* NPP001 (**C**) *P. autotrophica* NPP002 (**D**) Comparison of NPP B1 production yields. All production measurements were performed at least in duplicate.

As an alternative strategy to stimulate the production of NPP B1, pNPPB1s were transferred to the original NPP B1-producing strain, *P. autotrophica* GG5036SP via triparental conjugation [10]. The exconjugant strain containing 140 kb of pNPPB1s was named *P. autotrophica* NPP001 (Fig. 2B), but the production level of NPP B1 in *P. autotrophica* NPP001 was still unexpectedly low. Accordingly, pNPPREG2 with 32 kb of the right-end portion of NPP B1 BGC, which included the 8-kb deficiency gene of pNPPB1s, was overexpressed through the homologous recombination in *P. autotrophica* NPP001 and named *P. autotrophica* NPP002 (Fig. 2C). The *P. autotrophica* NPP002 strain consequently has two copies of the NPP B1 BGC. As a result, the production level of NPP B1 in the 2-L flask culture was 8.35 mg/l in *P. autotrophica* NPP002, which was increased approximately 7.3-fold compared to that produced by 1.15 mg/l in *P. autotrophica* GG5036SP strain (Fig. 2D).

### Transcription Analysis of NPP B1 BGC-Overexpressed Strains

**Fig. 3:**
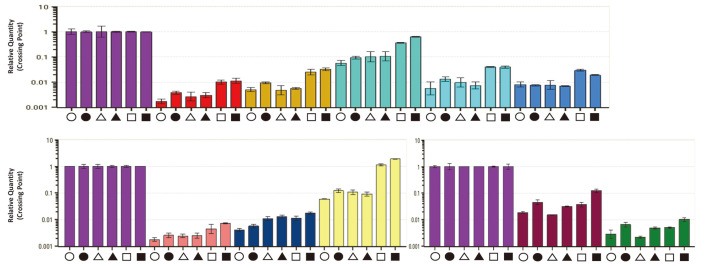
Transcription analysis of NPP B1 BGC by Real-Time qRT-PCR. Open circle, transcripts from the *P. autotrophica* GG5036SP mutant at 24 h; closed circle, transcripts from *P. autotrophica* GG5036SP mutant at 48 h; open triangle, transcripts from the *P. autotrophica* NPP001 at 24 h; closed triangle, transcripts from *P. autotrophica* NPP001 at 48 h; open quadrangle from the *P. autotrophica* NPP002 at 24 h; closed quadrangle, transcripts from *P. autotrophica* NPP002 at 48 h; house-keeping gene, *rpsO* (purple); PKS genes, *nppA* (red), *nppC* (yellow), *nppI* (emerald green) and *nppJ* (sky blue); regulatory genes related to NPP B1 biosynthesis, *nppRI* (blue), *nppRII* (pink), *nppRIII* (dark blue), *nppRIV* (light yellow), *nppRV* (brown) and *nppRVI* (green). All transcript measurements were performed at least in duplicate.

The transcription levels were compared to confirm the basis for the high level of NPP B1 production in the *P. autotrophica* NPP002 strain that contained two copies of a complete NPP B1 BGC. After sampling on days 1 and 2, transcript analysis was performed for four PKS genes (*nppA*, *nppC*, *nppI*, and *nppJ*) and six regulatory genes (*nppRI*, *RII*, *RIII*, *RIV*, *RV*, and *RVI*) of the NPP B1 BGC. The *P. autotrophica* NPP001 strain, containing only pNPPB1s, showed an overall similar transcription level to that of the *P. autotrophica* GG5036SP strain. In the case of *P. autotrophica* NPP002, a strain possessing the entire NPP B1 BGC, the overall transcription level, including the *nppA* gene, the starting module, increased. Among the regulators, *nppRIV* gene transcription increased remarkably compared to the other strains (Fig. 3). The *nppRIV* was proposed as a major NPP-specific positive regulatory gene present within the BGC, even though the function and mechanism of these six regulatory genes remain to be elucidated in greater detail [10].

### Potential RdRp Inhibition Activity of NPP B1

**Fig. 4:**
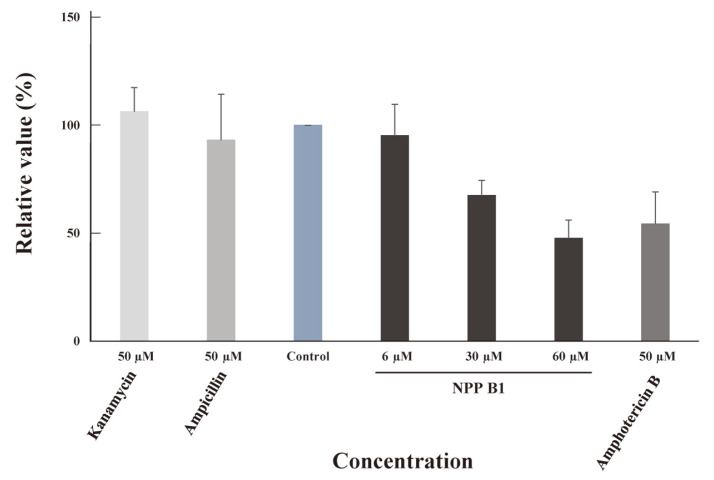
Comparison of RNA-dependent-RNA polymerase activities. The fluorescence emitted due to the synthesis of dsRNA in a reaction was recorded in the presence kanamycin (50 μM), ampicillin (50 μM), control (without antibiotics), NPP B1 (6 μM, 30 μM, 60 μM), or amphotericin B (50 μM). All assay measurements were performed at least in duplicate.

A fluorescence-based RdRp (RNA-dependent RNA polymerase) assay was performed to identify the antiviral activity of NPP B1. This analysis was based on the principle that when the dsRNA was synthesized from ssRNA by the RdRp of SARS-CoV-2, the fluorescence value increased over time with fluorescence dye, which can only bind to dsRNA. An enzyme-free version of the assay was set as a negative control under positive conditions to erase the background signal. In addition, when the compounds were treated by concentration to test under positive conditions, the same amount of compound was added to the negative control condition, which was set to background. The assay reaction time was 20 min. A comparison of how much the fluorescence decreased with the positive control at 10 min showed that NPP B1 had more than 50% inhibition than the positive control at 60-μM concentration, and amphotericin B showed more than 50% inhibition at 100-μM concentration. On the other hand, neither kanamycin, the aminoglycoside antibiotic, nor beta-lactam or ampicillin showed any noticeable inhibitory activity even at 100-μM concentration (Fig. 4).

## Discussion

NPP B1, which is a heptane compound with disaccharides, has a similar antifungal activity to that of amphotericin B, a strong antifungal agent. Despite the superior activity of NPP B1, it is challenging to develop as a drug because of the low titer. Here, to increase the productivity of NPP B1, NPP B1 BGC was isolated through the BAC library, and a 140-kb BAC plasmid (named pNPPB1s) with the NPP B1 BGC was obtained. On the other hand, pNPPB1s lacked approximately 8 kb of the right-end portion of the BGC, including some cluster-situated regulators. Although the BAC library is a useful method for isolating BGCs, it is still challenging to isolate an entire large-sized BGC without missing portions. The homologous and heterologous expression of pNPPB1s was attempted to increase its productivity. In the case of homologous overexpression, the production level was not increased in the *P. autotrophica* NPP001 strain. Interestingly, the production level was increased approximately 7.3-fold with *P. autotrophica* NPP002, which was also introduced with pNPPREG2 into the *P. autotrophica* NPP001 strain.

In our previous work, the pSBAC containing a tatutomycein (TMC) BGC named pMMBL101 was introduced into the TMC single copy-containing wild-type *Streptomyces* sp. CK4412 by conjugation [25]. Among the resulting ex-conjugants, four were randomly selected for further analysis and showed that pMMBL101 was integrated adjacent to the original TMC BGC in three of the four selected strains, whereas pMMBL101 inserted into the PhiBT1 attB site of the *Streptomyces* sp. CK4412 chromosome in only one strain [25]. These previous results suggested that the pSBAC system prefers homologous recombination rather than PhiBT1 site-specific attachment. pNPPREG, a pSBAC containing the 32-kb NPP-specific regulatory genes, was previously confirmed to be integrated into the NPP BGC in the chromosome via homologous recombination due to deletion of the attachment fragment present in the pSBAC [11].

Transcription analysis showed that *P. autotrophica* NPP 001 was similar to the parent strain *P. autotrophica* GG5036SP, but the overall transcription level in *P. autotrophica* NPP002 was increased. In particular, the expression level of the NPP RIV regulator was increased significantly. The NPP RIV regulator is believed to alleviate the limitation of NPP B1 productivity, since previous studies showed that the NPP pathway-specific regulatory genes affect productivity [26]. The NPP B1 productivity was increased approximately 5-fold when NPP pathway-specific regulatory genes (pNPPREG) were overexpressed in a strain with NTG random mutation [11]. In addition, pNPPREG also led to a significant increase in the yield of an NPP analogue named NPP B2 as well [6]. Since *P. autotrophica* NPP001 containing two copies of NPP biosynthetic genes in the chromosome failed to stimulate the NPP B1 titer (Fig. 2), overexpression of NPP pathway-specific regulatory genes seems to be more critical than the tandem repeat of NPP B1 biosynthetic genes missing the pathway-specific regulatory genes. In fact, the highest production level of NPP B1 ever achieved by *P. autotrophica* strains so far was approximately 31.6 mg/L via synergistic combination of the traditional random mutagenesis and pNPPREG chromosomal integration, which is higher than the value of 8.35 mg/L obtained in this study [11]. Although the BAC cloning of a large-sized NP BGC described here is a useful strategy for titer improvement and pathway refactoring in general, the tandem repeat of BAC-cloned NPP BGC in *P. autotrophica* exhibited no stimulatory effect on the NPP B1 titer. These results imply that there should be some unknown regulator(s) likely present only in *Pseudonocardia* species, which may play a critical regulatory role in NPP biosynthesis. Further research needs to be pursued to identify in more detail the relationship between global regulatory network and NPP-BAC clone expression in a rare actinomycetes like *P. autotrophica* [27, 28]. In addition, more comparative and systematic studies should be conducted to find out why NPP B1 production was not observed when pNPPB1s or pNPPB1s+pNPPREG was expressed in heterologous hosts such as *S. coelicolor* and *S. lividans* (data not shown). Moreover, co-expression of BAC-cloned NPP BGC and pNPPREG in the highest NPP B1-producting 3R-42 strain needs be pursued to further maximize the NPP B1 titer.

Finally, the increasing need for antiviral drugs prompted us to test the in vitro antiviral activity of NPP B1 using a fluorescence-based SARS-CoV-2 RdRp assay. Because RdRp is an essential enzyme involved in viral replication, it is difficult for a mutation to occur, so it is a good target for antiviral drugs. Fluorescence-based analysis confirmed that the fluorescence decreased with increasing NPP B1 concentration. Although the precise mechanism has not been elucidated, this highlights the potential of NPP B1 as a putative RdRp inhibitor. Finally, *Streptomyces* BAC cloning of a large-sized NP BGC is an ideal approach for stimulation and identification of various biological activities of secondary metabolites in actinomycetes.

## Supplemental Materials

Supplementary data for this paper are available on-line only at http://jmb.or.kr.

## Figures and Tables

**Table 1:** Bacterial strains and plasmids used in this study.

Strain/plasmid	Relevant characteristics	Source/reference
Plasmid		
pESAC13	Vector used for library construction, *aacIII(IV)*, *oriT*, *attP-int*	Bio S&T
pNPPB1s	pESAC13 with NPP B1 BGC excluding *nppO*, *nppM*, and three regulatory genes (*nppRIV*, *nppRV*, *nppRVI*)	This work
pUN8	pUC19 with 8 kb of the right-hand portion of NPP BGC (*nppO*-*nppRVI*)	This work
pSEN8	pSE34 with *oriT* and 8 kb of the right-hand portion of NPP BGC (*nppO*-*nppRVI*) containing KanR	This work
pNPPREG	pSBAC with 32 kb of the right-hand portion of BGC (part of *nppC*-*nppRVI*)	[11]
pNPPREG2	pNPPREG, which replaced *Apr^R^* with *Hyg^R^*	This work
pNPPREG3	pNPPREG, which replaced *Apr^R^* with *Kan^R^*	This work
*E. coli*		
DH10B	*E. coli* host for cloning and constructs derived from various BAC vectors	Bio S&T
DH5α	*E. coli* host for cloning and constructs derived from various vectors	TRANS
EPI300	*E. coli* host for cloning and constructs derived from various vectors	
ET12567/pUB307	*E. coli* host for transferring various plasmids into *Streptomyces* via conjugation	
*Pseudonocardia autotrophica*		
KCTC9441	Original NPP A1-producing strain	
GG5036SP	NPP B1-producing strain (mutation in *nppC*)	[10]
NPP001	GG5036SP with pNPPB1s	This work
NPP002	NPP001 with pNPPREG2	This work
