# Supplementary material for: Streptomyces BAC Cloning of a Large-Sized Biosynthetic Gene Cluster of NPP B1, a Potential SARS-CoV-2 RdRp Inhibitor
Source: J Microbiol Biotechnol. 2022 Jun 13;32(7):911–7. doi: 10.4014/jmb.2205.05036 (PMC9628923; doi:10.4014/jmb.2205.05036)
Supplement: Supplementary file 1 [file jmb-32-7-911-supple.pdf]

J. Microbiol. Biotechnol.
https://doi.org/10.4014/jmb.2205.05036

Table S1. Primer list used in this study

Primer	Primer sequence, 5′→3′	Purpose
*nppA*_F	GGCCACTCCATCGGTGAGAT	qRT-PCR
*nppA*_R	GTAGCGCCTGCATGAGTGTG	qRT-PCR
nppB F	CCGTTCTACTCCACCGTCA	qRT-PCR
nppB R	TGACCTCCACGAAGATCCG	qRT-PCR
*nppC*_F	GAGTACCGGAACCGGCTCAA	qRT-PCR
*nppC*_R	GGGAAGTCGAACAGCAGGGT	qRT-PCR
*nppI*_F	TCCGCATCCTCACCGAGAAC	qRT-PCR
*nppI*_R	CGATGATGGTGTGGGCGTTG	qRT-PCR
nppJ F	CCGGCTTCTTCGGTATCTCA	qRT-PCR
nppJ R	CCAGGTAGGCGTAGTCCTG	qRT-PCR
*nppRI*_F	CGGACCGGACAACCTCTACC	qRT-PCR
*nppRI*_R	CAGCAGGAACCCGAGGAAGG	qRT-PCR
*nppRII*_F	GACGTCCTGCACGAGTGGT	qRT-PCR
*nppRII*_R	CACTGTCCCGTGTCCTCGTC	qRT-PCR
*nppRIII*_F	CTGGAGTGCCTGCTCGACTG	qRT-PCR
*nppRIII*_R	CTCCTCGTCGGCCCATTCC	qRT-PCR
*nppRIV*_F	GCCTCCACCCTGTTCCTCAG	qRT-PCR
*nppRIV*_R	CCACGAACCCAGCTCGAAGA	qRT-PCR
*nppRV*_F	GGCCTGACCAACCACGAGAT	qRT-PCR
*nppRV*_R	AGGCGATACGATCGGAACCA	qRT-PCR
*nppRVI*_F	CCTGGCGGTGGAACTACCTG	qRT-PCR
*nppRVI*_R	ACGATCCCGACAGCCATCAG	qRT-PCR

nppY F	CTTCACTGCCGAACTCCGCCAGTA	check for NPP BGC
nppY R	TGAAGGTGGTGCCCTGGGTCCATT	check for NPP BGC
nppN F	TCGAAGGCGTCGTCGTCGTGGTTG	check for NPP BGC
nppDII(N) R	TGAGCCGCCAGCCGATGTACCTCGA	check for NPP BGC
nppRVI check F	ACATCGCCTGATGGCTGTCGGGAT	check for NPP BGC
nppRVI check R	AGGTCTTCCGGTGGGCCTCGGTCTT	check for NPP BGC
8kb infusion F	GACCGCCGGCTCTAGTCTAGAAGTTCCCGCCAGCC	Construction of pSE34_8kb
8kb infusion R	GGCAACCCGAGCTAGGAATTCACTGGCCGTCGTTTTAC	Construction of pSE34_8kb
Kan _F	GGTGGAGTGCAAGCTAGCTTCACGCTGCCGCAAGC	kan^R PCR for pSEN8
Kan_R	TGATTACGCCAAGCTAATCGAAATCTCGTGATGGC	kan^R PCR for pSEN8
Ch_kan F	ACCTCTGACTTGAGCGTCGATT	Check for insertion of kan^R and hyg^R
Ch_kan R	AGGCTTTTTCATATCTCA	Check for insertion of kan^R
Ch_hyg R	ATTTTCGTTAGTCGGAGGC	Check for insertion of hyg^R
hyg_F	TGTCATCAGCGGTGGAGTGCAATGTCGTGCAATACGAATGA GAACCAGGCGGTGGCGTAC	hyg^R PCR for BAC modification
hyg_R	TCTGGCGGATGCAGGAAGATCAACGGATCTCGGCCCAGTT GAGTTCTCCGCTCATGAGAAC	hyg^R PCR for BAC modification
